# Supplementary material for: Heat adaptation of phage T7 under an extended genetic code
Source: Virus Evol. 2021 Dec 1;7(2):veab100. doi: 10.1093/ve/veab100 (PMC8923235; doi:10.1093/ve/veab100)
Supplement: veab100_Supp [file veab100_supp.zip › Sequencing_Analysis.zip!/Sequencing_Analysis/S1/evidence/RA_14.html]

BRESEQ :: Evidence


| Read alignment evidence... | | | | | | | | | | | |
| --- | --- | --- | --- | --- | --- | --- | --- | --- | --- | --- | --- |
|  | seq id | position | | ref | new | freq | score (cons/poly) | reads | annotation | genes | product |
| \* | NC\_001604.1 | 13,566 | 1 | . | A | 33.4% | 5269.3 / inf | 4545 | intergenic (–/–) | *–/–* | –/– |
| Reads supporting (aligned to +/- strand):  **ref** base . (1447/1576);  **new** base A (717/801);  **total** (2165/2380) | | | | | | | | | | | |
| Fisher's exact test for biased strand distribution *p*-value = 7.06e-01 | | | | | | | | | | | |
| Kolmogorov-Smirnov test that lower quality scores support variant *p*-value = 1.60e-06 | | | | | | | | | | | |

Only 100 of 4662 total aligned reads displayed.  

`CAGGATTCTTGATGACTTTCCAGACGACTACGAGAAGTTTCGCTGGAGAGTCCCATTCTAATACGACTCACTAAAGGAGACACACCATGTTCAAACTGATTAAGAAGTTAGGCCAACTGCTGGTTCGTATGTACAACGTGGAAGCCAAGCGACTGAACGATGAGGCTCGTAAAGAGGCCACACAGTCACGCGCTCTGGCGATTCGCTCCAACGAACTGGCTGACAGTGCATCCACTAAAGTTACCGAGGCTGCCCGTGTGGCAAACCAAGCTCAACAGCTTTCCAAATTCTTTGAGTAATC‑AAACAGGAGAAACCATTATGTCTAACGTAGCTGAAACTATCCGTCTATCCGATACAGCTGACCAGTGGAACCGTCGAGTCCACATCAACGTTCGCAACGGTAAGGCGACTATGGTTTACCGCTGGAAGGACTCTAAGTCCTCTAAGAATCACACTCAGCGTATGACGTTGACAGATGAGCAAGCACTGCGTCTGGTCAATGCGCTTACCAAAGCTGCCGTGACAGCAATTCATGAAGCTGGTCGCGTCAATGAAGCTATGGCTATCCTCGACAAGATTGATAACTAAGAGTGGTATCCTC  >  NC_001604.1/13266‑13866`  

                                                                                                                                                                                                                                                                                                             `|`                                                                                                                                                                                                                                                                                                              
      `tCTTGATTACTTTCCAGACGACTACGAGAAGTTTCGCTGGAGAGTCCCATTCTAATACGACTCACTAAAGGAGACACACCATGTTCAAACTGATTAAGAAGTTAGGCCAACTGCTGGTTCGTATGTACAACGTGGAAGCCAAGCGACTGAACGATGAGGCTCGTAAAGAGGCCACACAGTCACGCACTCTGGCGATTCGCTCCAACGAACTGGCTGACAGTGCATCCACTAAAGTTACCGAGGCTGCCCGTGTGGCAAACCAAGCTCAACAGCTTTCCAAATTCTTTGAGTAATC‑AAtagg                                                                                                                                                                                                                                                                                                        <  1:110685‑M1/301‑5 (MQ=255)`  
      `tCTTAATGACTTTCCAGACGACTACGAGAAGTTTCGCTGGAGAGTCACAGTTTAATACGAATCACTAAAGAGGACACACCATGTTCAAACTGATTAAGAAGTTAGGCAAAATGCTGGTTCGTATGTACAACGTGGAAGCCAAGCGACTGAACGATAAGGTTCGTAAAGAGGCCACACAGTCACGCGCTCTGGCGATTCGCTCCAACGAACTGGCTGACAGTGCATCCACTAAAGTTACCGAGGCTGCCCGTGTGGCAAACCAAGCTCAACAGCTTTCCAAATTCTTTGAGTAATC‑AAtagg                                                                                                                                                                                                                                                                                                        <  1:483204‑M1/301‑5 (MQ=255)`  
        `ttGATGAGTTTCCAGACGACAACGAGAAGTTTCGCTGGAGAGTCCCATTCTAATACGAATCACTAAAGGAGACACACCATGTTCAAACTGATTAAGAAGTTAGTCCAACTGCTGGTTCGTATGTACACCGTGGAAGCCAAGCGACTTCACGATGAGGCTCGTAAAGAGGCCACACAGTCACGCGCTCTGGCGATTCGCTCCAACGAACTGGCTGACAGTGCATCCACTAAAGTTACCGAGGCTGCCCGTGTGGCAAACCAAGCTCAACAGCTTTCCAAATTCTTTGAGTAATC‑AAtaggag                                                                                                                                                                                                                                                                                                      <  1:630822‑M1/301‑7 (MQ=255)`  
             `gaCTTTCCAGACGACTACGAGAAGTTTCGCTGGAGAGTCCCATTCTAATACGACTCACTAAAGGAGACACACCATGTTCAAACTGATTAAGAAGTTAGGCCAACTGCTGGTTCGTATGTACAACGTGGAAGCCAAGCGACTGAACGATGAGGCTCGTAAAGAGGCCACACAGTCACGCGCTCTGGCGATTCGCTCCAACGAACTGGCTGACAGTGCATCCACTAAAGTTACCGAGGCTGCCCGTGTGGCAAACCAAGCTCAACAGCTTTCCAAATTCTTTGAGTAATC‑AAtaggagaaatc                                                                                                                                                                                                                                                                                                 <  1:208716‑M1/301‑12 (MQ=255)`  
                `tttCCAGACGACTACGAGAAGTTTCGCTGGAGAGTCCCATTCTAATACGACTCACTAAAGGAGACACACCATGTTCAAACTGATTAAGAAGTTAGGCCAACTGCTGGTTCGTATGTACAACGTGGAAGCCAAGCGACTGAACGATGAGGCTCGTAAAGAGGCCACACAGTCACGCGCTCTGGCGATTCGCTCCAACGAACTGGCTGACAGTGCATCCACTAAAGTTACCGAGGCTGCCCGTGTGGCAAACCAAGCTCAACAGCTTTCCAAATTCTTTGAGTAATC‑AAtaggagaaatcaaa                                                                                                                                                                                                                                                                                              >  1:429432‑M1/1‑287 (MQ=255)`  
                   `ccAGACGACTACGAGAAGTTTCGCTGGAGAGTCCCATTCTAATACGACTCACTAAAGGAGACACACCATGTTCAAACTGATTAAGAAGTTAGGCCAACTGCTGGTTCGTATGTACAACGTGGAAGCCAAGCGACTGAACGATGAGGCTCGTAAAGAGGCCACACAGTCACGCGCTCTGGCGATTCGCTCCAACGAACTGGCTGACAGTGCATCCACTAAAGTTACCGAGGCTGCCCGTGTGGCAAACCAAGCTCAACAGCTTTCCAAATTCTTTGAGTAATC‑AAACAGGAGAAATCAatat                                                                                                                                                                                                                                                                                           >  1:424167/1‑301 (MQ=255)`  
                           `cTACGAGAAGTTTCGCTGGAGAGTCCCATTCTAATACGACTCACTAAAGGAGACACACCATGTTCAAACTGATTAAGAAGTTAGGCCAACTGCTGGTTCGTATGTACAACGTGGAAGCCAAGCGACTGAACGATGAGGCTCGTAAAGAGGCCACACAGTCACGCGCTCTGGCGATTCGCTCCAACGAACTGGCTGACAGTGCATCCACTAAAGTTACCGAGGCTGCCCGTGTGGCAAACCAAGCTCAACAGCTTTCCAAATTCTTTGAGTAATCAAAcaggagaaatcaatatgatcgttt                                                                                                                                                                                                                                                                                    >  1:100160‑M1/1‑277 (MQ=255)`  
                               `gagaAGTTTCGCTGGAGAGTCCCATTCTAATACGACTCACTAAAGGAGACACACCATGTTCAAACTGATTAAGAAGTTAGGCCAACTGCTGGTTCGTATGTACAACGTGGAAGCCAAGCGACTGAACGATGAGGCTCGTAAAGAGGCCACACAGTCACGCGCTCTGGCGATTCGCTCCAACGAACTGGCTGACAGTGCATCCACTAAAGTTACCGAGGCTGCCCGTGTGGCAAACCAAGCTCAACAGCTTTCCAAATTCTTTGAGTAATC‑AAtaggagaaatcaatatgatcgtttctgac                                                                                                                                                                                                                                                                               <  1:536260‑M1/301‑30 (MQ=255)`  
                                        `cGCTGGAGAGTCCCATTCTAATACGACTCACTAAAGGAGACACACCATGTTCAAACTGATTAAGAAGTTAGGCCAACTGCTGGTTCGTATGTACAACGTGGAAGCCAAGCGACTGAACGATGAGGCTAGTAAAGAGGCCACACAGTCACGCGCTCTGGCGATTCGCTCCAACGAACTGGCTGACAGTGCATCCACTAAAGTTACCGAGGCTGCCCGTGTGGCAAACCAAGCTCAACAGCTTTCCAAATTCTTTGAGTAATCAAAcaggaaaaatcaatatgatcgtttctgacatcgaagc                                                                                                                                                                                                                                                                       >  1:85908‑M1/1‑264 (MQ=255)`  
                                                 `gTCCCATTCTAATACGACTAACTAAAGGAGACACACCATGTTCAAACTGATTAAAAAGTTAGGCCAACTGCTGGTTCGTATGTACAACGTGGAAGCCAAGCGACTGAACGATGAGGCTCGTAAAGAGGCCACACAGTCACGCGCTCTGGCGATTCGCTCCAACGAACTGGCTGACATTGCATCCAATAAAGTTACCGAGGCTGCCCGTGTGGCAAACCAAGCTCAACAGCTTTCCAAATTCTTTGAGTAATC‑AAtaggagaaatcaatatgatcttttctgacctcgaaactaacgccctt                                                                                                                                                                                                                                                             >  1:375798‑M1/1‑254 (MQ=255)`  
                                                      `gttcTAATACGACACACTAAAGAAGACACACCATGTTCAAACTGATTAAGAAGTTAGGCCAACTGCTGGTTCGTATGTACAACGTGGAAGCCAAGCGACTGAACGATGAGGCTCGTAAAGAGGCCACACAGTCACGCGCTCTGGCGATTCGCTCCAACGAACTGGCTGACAGTGCATCCACTAAAGTTACCGAGGCTGCCCGTGTGGCAAACCAAGCTCAACAGCTTTCCAAATTCTTTGAGTAATCAAAcaggagaaatcaatatgatcgtttctgacatcgaagctaacgccctcttag                                                                                                                                                                                                                                                         <  1:406381‑M1/300‑52 (MQ=255)`  
                                                      `aTTCTAATACGACTCACTAAAGGAGACACACCATGTTCAAACTGATTAAGAAGTTAGGCCAACTGCTGGTTCGTATGTACAACGTGGAAGCCAAGCGACTGAACGATGAGGCTCGTAAAGAGGCCACACAGTCACGCGCTCTGGCGATTCGCTCCAACGAACTGGCTGACAGTGCATCCACTAAAGTTACCGAGGCTGCCCGTGTGGCAAACCAAGCCCAACAGCATGCCAAATTCTTTGAGTAATCAAAcaggagaaaacaaaaggatcgtttctgacatcgaagctaacgccctcttag                                                                                                                                                                                                                                                         >  1:220366‑M1/1‑250 (MQ=255)`  
                                                       `ttCTAATACGACTCACTAAAGGCGACACACCATGTTCAAACTGATTAAGAAGTTAGGCCAACTGCTGGTTCGTATGTACAACGTGGAAGCCAAGCGACTGAACGATGAGGCTCGTAAAGAGGCCACACAGTCACGCGCTCTGGCGATTCGCTCCAACGAACTGGCTGACAGTGCATCCACTAAAGTTACCGAGGCTGCCCGTGTGGCAAACCAAGCTCAACAGCTTTCCAAATTCTTTGAGTAAAC‑AAtaggagaaatcaatatgatcgtttctgacatcgaagctaacgccctcttagag                                                                                                                                                                                                                                                       <  1:380794‑M1/301‑54 (MQ=255)`  
                                                       `ttCTAATACGACTCACTAAAGGAGACACACCATGTTCAAACTGATTAAGAAGTTAGGCCAACTGCTGGTTCGTATGTACAACTTGGAAGCCAAGCGACTGAACGATGAGGCTCGTAAAGAGGCCACACAGTCACGCGCTCTGGCGATTCGCTCCAACGAACTGGCTGACAGTGCATCCACTAAAGTTACCGATGCTGCCCGTGTGGCAAACCAAGCTCAACAGCTTTCCAAATTCTTTGAGTAATCAAAcatgagacatcaatatgatcgtttctgacatcgaatctaacgccctcttcta                                                                                                                                                                                                                                                        >  1:472229‑M1/1‑249 (MQ=255)`  
                                                       `ttCTAATACGACTCACTAAAGGAGACACACCATGTTCAAACTGATTAAGAAGTTAGGCCAACTGCTGGTTCGTATGTACAACGTGGAAGCCAAGCGACTGAACGATGAGGCTCGTAAAGAGGCCACACAGTCACGCGCTCTGGCGATTCGCTCCAACGAACTGGCTGACAGTGCATCCACTAAAGTTACCGAGGCTGCCCGTGTGGCAAACCAAGCTCAACAGCTTTCCAAATTCTTTGAGTAATCAAAcaagagaaatcaatatgatcgtttctgacatcgaagctaacgccctcttaga                                                                                                                                                                                                                                                        >  1:196893‑M1/1‑249 (MQ=255)`  
                                                       `ttCTAATACGACTCACTAAAGGAGACACACCATGTTCAAACTGATTAAGAAGTTAGGCCAACTGCTGGTTCGTATGTACAACGTGGAAGCCAAGCGACTGAACGATGAGGCTCGTAAAGAGGCCACACAGTCACGCGCTCTGGCGATTCGCTCCAACGAACTGGCTGACAGTGCATCCACTAAAGTTACCGAGGCTGCCCGTGTGGCAAACCAAGCTCAACAGCTTTCCAAATTCTTTGAGTAATC‑AAtaggagaaatcaatatgatcgtttctgacatcgaagctaacgccctcttagag                                                                                                                                                                                                                                                       <  1:353173‑M1/301‑54 (MQ=255)`  
                                                       `ttCTAATACGACTCACTAAAGGAGACACACCATGTTCAAACTGATTAAGAAGTTAGGCCAACTGCTGGTTCGTATGTACAACGTGGAAGCCAAGCGACTGAACGATGAGGCTCGTAAAGAGGCCACACAGTCACGCGCCCTGGCGATTCGCTCCAACGAACTGGCTGACAGTGCATCCACTAAAGTTACCGAGGCTGCCCGTGTGGCAAACCAAGCTCAACAGCTTTCCAAATTCTTTGAGTAATCAAAcaggagaaatcaatatgatcgtttctgacatcgaagctaacgccctcttaga                                                                                                                                                                                                                                                        >  1:500963‑M1/1‑249 (MQ=255)`  
                                                       `ttCTAATACGACTCACTAAAGGAGACACACCATGTTCAAACTGATTAAGAAGTTAGGCCAACTGCTGGTTCGTATGTACAACGTGGAAGCCAAGCGACTGAACGATGAGGCTCGTAAAGAGGCCACACAATCACGCGCTCTGGCGATTCGCTCCAACGAACTGGCTGACAGTGCATCCACTAAAGTTACCGAGGCTGCCCGTGTGGCAAACCAAGCTCAACAGCTTTCCAAATTCTTTGAGTAATC‑AAtaggagaaatcaatatgatcgtttctgacatcgaagctaacgccctcttagag                                                                                                                                                                                                                                                       <  1:569359‑M1/301‑54 (MQ=255)`  
                                                       `ttCTAATACGACTCACTAAAGGAGACACACCATGTTCAAACTGATTAAGAAGTTAGGCCAACTGCTGGTTAGTATGTACAACGTGGAAGCCAAGCGACTGAACGATGAGGCTCGTAAAGAGGCCACACAGTCACGCGCTCTGGCGATTCGCTCCAACGAACTGGCTGACAGTGCATCCACTAAAGTTACCGAGGCTGCCCGTGTGGCAAACCAAGCTCAACAGCTTTCCAAATTCTTTGAGTAATC‑AAtaggagaaatcaatatgatcgtttctgacatcgaagctaacgccctcttagag                                                                                                                                                                                                                                                       <  1:225512‑M1/301‑54 (MQ=255)`  
                                                       `ttCAAATACGACTCACTAAAGGAGACAAACCAAATTCAAACTGATTAAGAAGTTAGGCCAACTGCTGGTTCGTATGTAAAACGTGGAAGCCAAGCGACTGAACGATGAGGCTCGGAAAGAGGCCACACAGTCACGCGCTCTGGCGATCCGCTCCAACGAACTGGCTGACAGTGCATCCACTAAAGTTACCGAGGCTGCCCGTGTGGCAAACCAAGCTCAACAGCTTTCCAAATTCTTTGAGTAATC‑AAgaggagaaatcaatatgatcgtttctgacatcgaagctaacgccctcttagag                                                                                                                                                                                                                                                       <  1:327377‑M1/301‑54 (MQ=255)`  
                                                       `taagagacagactcactaaAGGAGACACACCATGTTCAAACTGATTAAGAAGTTAGGCCAACTGATGGTTCGTATGTACAACGTGGAAGCCAAGCGACTGAACGATGAGGCTCGTAAAGAGGCCACACAGTCACGCGCTCTGGCGATTCGCTCCAACGAACTGGCTGACAGTGCATCCACTAAAGTTACCGAGGCTGCCCGTGTGGCAAACCAAGCTCAACAGCTTTCCAAATTCTTTGAGTAATC‑AAtaggagaaatcaatatgatcgtttctgacatcgaagctaacgccctcttagag                                                                                                                                                                                                                                                       <  1:419171‑M1/292‑54 (MQ=255)`  
                                                       `gtataagagacagaactaaaggagacaaaCCATGTTCAAACTGATTAAGAAGTTAGGCCAACTGCTGGTTCGTATGTAAAAAGGGGAAGCCAAGCGACTGAACGATGAGGCTCGTAAAGAGGCAACACAGTCACGCGCTCTGGCGATTCGCTACAACGAACTGGCTGACAGTGCATCCACTAAAGTTACCGAGGCTGCCCGTGTGGCAAACCAAGCTCAACAGCTTTCCAAATTCTTTGAGTAATC‑AAtaggagaaatcaatatgatcgtttctgacatcgaagctaacgccctcttagag                                                                                                                                                                                                                                                       <  1:159738‑M1/287‑54 (MQ=255)`  
                                                       `aaggaatactACTCACAAAAGGAGACAAACCAAGTTCAAACTGAGTAAGAAGGGAGGCCAAATGCGGGTTCGTATGTACAACCGGGAAGCCAAGCGACTGAACGATGAGGCGCGGAAAGAGGCCACACAGTCACGCGCTCTGGCGATTCGGGCCAACGAACTGGCTGACAGGGCATCCACTAAAGTGACCGAGGCGGCCCGTGTGGCAAAACAAGCTCAACAGCTTTCCAAATTCTGTGAGTAAGC‑AAtaggagaaatcaatatgatcgtttatgacatcgaagctaacgccctcttagag                                                                                                                                                                                                                                                       <  1:395347‑M1/297‑54 (MQ=255)`  
                                                        `tataagagacagcactaaaggagacACACAATGTTCAAACTGATTAAGAAGTTAGGCCAACTGCTGGTTCGTATGTACAACGTGGAAGCCAAGCGACTGAACGATGAGGCTCGTAAAGAGGCCACACAGTCACGCGCTCTGGCGATTCGCTCCAACGAACTGGCTGACAGTGCATCCACTAAAGTTACCGAGGCTGCCCGTGTGGCAAACCAAGCTCAACAGCTTTCCAAATTCTTTGAGTAATCAAAcaggagaaatcaatatgatcgtttctgacatcgaagctaacgccctcttagag                                                                                                                                                                                                                                                       <  1:27967‑M1/289‑54 (MQ=255)`  
                                                        `tCTAATACGACTCACTAAAGGAGACACACCATGTTCAAACTGATTAAGAAGTTAGGCCAACTGCTGGTTCGTATGTACAACGTGGAAGCCAAGCGACTGAACGATGAGGCTCGTAAAGAGGCCACACAGTCACGCGCTCTGGCGATTCGCTTCAACGAACTGGCTGACAGTGCATCCACTAAAGTTACCGAGGCTGCCCGTGTGGCAAACCAAGCTCAACAGCTTTCCAAATTCTTTGAGTAATCAAAcaggagaaatcaatatgatcgtttctgacatcgaagctaacgccctcttagag                                                                                                                                                                                                                                                       <  1:636032‑M1/301‑54 (MQ=255)`  
                                                        `aagaagagacagcaataaaggagacacaccaTGTTCAACCTGATTAAGAAGTTAGGCCAACTGCTGGTTCGTATTTACAACGTGGAAGCCAAGCGACTGAACGATGAGGCTCGTAAAGATGCCACACAGTAACGCGCTCTGGCGATTCGCTCCAACGAACTGGCTGACAGTGCATCCACTAAAGTTACCGAGGCTGCCCGTGTGGCAAACCAAGCGCAACAGCTTTCCAAATTCTTTGAGTAATCAAAcaggagaaatcaatatgatcgtttctgacatcgaagctaacgccctcttagag                                                                                                                                                                                                                                                       <  1:203604‑M1/286‑54 (MQ=255)`  
                                                         `tataaagagacagctaaaggagacacaCCATGTTCAAACTGATTAAGAAGTTAGGCCAACTGCTGGTTCGTATGTACAACGTGGAAGCCAAGCGACTGAACGATGAGGCTCGTAAAGAGGCCTCACAGTCACGCGCTCTGGCGATTCGCTCCAACGAACTGGCTGACAGTGCATCCACTAAAGTTACCGAGGCTGCCCGTGTGGCAAACCAAGCTCAACAGCTTTCCAAATTCTTTGAGTAATC‑AAtaggagaaatcaatatgatcgtttctgacatcgaagctaacgccctcttagagag                                                                                                                                                                                                                                                     <  1:79568‑M1/288‑56 (MQ=255)`  
                                                         `cttatacTACTCAATAAAAGAGACACGCCATGTTCAAACAGATTAAGAAGTTAGGCCAACCGCTGGTTCCTATTTACCACGTGGAAGCCAAGCGACTGAACGATGACGGTCGTAAAGCGGCCACACCGTCACGCGCTCTGGCGATTCGCTCCAACGAACTGGCTGACAGTGCTTCCACTAAAATTTCCGAGGCTGCCCGTGTGGCAAACCCAGCTCAACAGCTTTCCAACTTCTTTGAGTAATC‑AAtaggagaaatcaatatgatcgtttctgacatcgaagctaacgccctcttagagag                                                                                                                                                                                                                                                     <  1:446919‑M1/298‑56 (MQ=255)`  
                                                               `cGACTCACTAAAAGAGACACAGCATGTTCAAACTGATTAAGAAGTTAGGCCAACTGCTGGTTCGGATGAACATCGTCGAAGCCAAGCGACTGAACGATGAGGCTCGTAAAGAGGCCACACAGTCACGCGCTCTGGCGATTCGCGCCAACGAACTGGCTAACATTGCATCCACTAAAGTTACCGAGGCTGCCCGTGTGGCAAACCAAGCTCAACAGCTTTCCAAATTCTTTGAGTAATC‑AAtatgagaaatcaatatgatcgtttctgacatcgaagctaacgccctcttagagagcgtcac                                                                                                                                                                                                                                               <  1:266697‑M1/301‑62 (MQ=255)`  
                                                                `gACTCACTAAAGGAGACACACCATGTTCAAACTGATTAAGAAGTTAGGCCAACGGCTGGTTCGTATGTACAACGTGGAAGCCAAGCGACTGAACGATGAGGCTCGTAAAGAGGCCACACAGTCACGCGCTCTGGCGATTCGCTCCAACGAACTGGCTGACAGTGCATCCACTAAAGTTACCGAGGCTGCCCGTGTGGCAAACCAAGCTCAACAGCTTTCCAAATTCTTTGAGTAATCAAAcaggagaaatcaatatgatcgtttctgacatcgaagctaacgccctcttagagagcgtcac                                                                                                                                                                                                                                               <  1:440910‑M1/301‑62 (MQ=255)`  
                                                                   `tCACTAAAGGAGACACACCATGTTCAAACTGATTAAGAAGTTAGGCCAACTGCTGGTTCGTATGTACAACGTGGAAGCCAAGCGACTGAACGATGAGGCTCGTAAAGAGGCCACACAGTCACGCGCTCTGGCGATTCGCTCCAACGAACTTGCTGACAGTGCATCCACTAAAGTTACCGAGGCTGCCAGTGTGGCAAACCAAGCTCCACAGCTTTTCAACTTCCTTGAGTAATCAAAccggagaaatcaatatgatcgtttctgacatcgacgctaacgccctcttaaagagcttcactaa                                                                                                                                                                                                                                            >  1:315250‑M1/1‑237 (MQ=255)`  
                                                                    `cACTAAAGGAGACACACCATGTTCAAACTGATTAAGAAGTTAGGCCAACTGCTGGTTCGTATGTACAACGTGGAAGCCAAGCGACTGAACGATGAGGCTCGTAAAGAGGCCACACAGTCACGCGCTCTTGCGATTCGCTCCAACGAACTGGCTGACAGTGCATCCACTAAAGTTACCGAGGCTGCCCGTGTGGCAAACCAAGCTCAACAGCTTTCCAACTTCTTTGAGTAATC‑AAtaggagaaatcaacatgatcgtttctgacatcgaaactaacgccatcttagtgagcgtcaccattt                                                                                                                                                                                                                                          >  1:115624‑M1/1‑235 (MQ=255)`  
                                                                    `cACTAAAGGAGACACACCATGTTCAAACTGATTAAGAAGTTAGGCCAACTGCTGGTTCGTATGTACAACGTGGAAGCCAAGCGACTGAACGATGAGGCTCGTAAAGAGGCCACACAGTCACGCGCTCTGGCGATTCGCTCCAACGAACTGGCTGACAGTGCATCCACTAAAGTTACCTAGGCTGCCCGTGTGGCAAACCAAGCTCAACAGCTTTCCAAATTCTTTGAGTAATCAAAcaggagaaatcaatatgatcgtttctgacatcgaagctaacgccctcttagagagcgtcactaag                                                                                                                                                                                                                                           >  1:609614‑M1/1‑236 (MQ=255)`  
                                                                    `cACTAAAGGAGACACACCATGTTCAAACTGATTAAGAAGTTAGGCCAACTGCTGGTTCGTATGTACAACGTGGAAGCCAAGCGACTGAACGATGAGGCTCGTAAAGAGGCCACACAGTCACGCGCTCTGGCGATTCGCTCCAACGAACTGGCTGACAGTGCATCCACTAAAGTTACCGAGGCTGCCCGTGTGGCAAACCAAGCTCAACAGCTTTCCAAATTCTTTGAGTAATCAAAcaggagaaatcaatatgatcgtttctgacatcgaagctaacgccctcttagagagcgtcactaag                                                                                                                                                                                                                                           >  1:615009‑M1/1‑236 (MQ=255)`  
                                                                           `ggAGACACACCATGTTCAAACTGATTAAGAAGTTCGGCCAACTGCTGGTTCGTATGTACAACGTGGAAGCCAAGCGACTGAACGATGAGGCTCGTAAAGAGGCCACACAGTCACGCGCTCTGGCGATTCGCTCCAACGAACTGGCTGACAGTGCATCCACCAAAGTTACCGAGGCTGCCCGTGTGGCAAACCAAGCTCAACAACTTTCCAAATTCTTTGAGTAATCAAAcagaagaaataaatatgatcgtttctgacatagaagctaacgccctcttagagagcgtaactaagttccacc                                                                                                                                                                                                                                    >  1:7409‑M1/1‑229 (MQ=255)`  
                                                                           `ggAGACACACCATGTTCAAACTGATTAAGAAGTTAGGCCAACTGCTGGTTCGTATGTACAACGTGGAAGCCAAGCGACTGAACGATGAGGCTCGTAAAGAGGCCACACAGTCACGCGCTCTGGCGATTCGCTCCAACGAACTGGCTGACAGTGCATCCACTAAAGTTACCGAGGCTGCCCGTGTGGCAAACCAAGCTCAACAGCTTTCCAATTTCTTTGAGTAATC‑AAtaggagaaatcaatatgatcgtttctgacatcgaagctaacgccctcttagagagcgtcactaagttccactt                                                                                                                                                                                                                                   >  1:178274‑M1/1‑228 (MQ=255)`  
                                                                           `ggAGACACACCATGTTCAAACTGATTAAGAAGTTAGGCCAACTGCTGGTTCGTATGTACAACGTGGAAGCCAAGCGACTGAACGATGAGGCTCGTAAAGAGGCCACACAGTCACGCGCTCTGGCGATTCGCTCCAACGAACTGGCTGACAGTGCATCCACTAAAGTTACCGAGGCTGCCCGTGTGGCAAACCAAGCTCAACAGCTTTCCAAATTCTTTGAGTAATCAAAcaggagaaatcaatatgatcgtttctgacatcgaagctaacgccctcttagagagcgtcactaagttccact                                                                                                                                                                                                                                    >  1:66674‑M1/1‑229 (MQ=255)`  
                                                                                `aacaccATGTTCAAACTGATTAAGAAGTTAGGCCAACTGCTGGTTCGTATGTACAACGTGGAAGCCAAGCGACTGAACGATGAGGCTCGTAAAGAGGCCACACAGTCATGCGCTCTGGCGATTCGCTCCAACGAACTGGCTGACAGTGCATCCACTAAAGTTACCGAGGCTGCCCGTGTGGCAAACCAAGCTCAACAGCTTTCCAAATTCTTTGAGTAATC‑AAtaggagaaatcaatatgatcgtttctgacatcgaagctaacgccctcttagagagcgtcactaagttccactgcgggg                                                                                                                                                                                                                              <  1:273091‑M1/300‑79 (MQ=255)`  
                                                                                           `caatgagtttaggaagttAGGCCAACTGCTGTCACATATGAACAACGTGGAAGCCAACGCACTGTTCGATGCGGTTAGTAAAGATGCAACACAGTCACGCGATTTGGCGATTCGCTGCAACGAACTGGCTGACAGTGCCTCCTCTAAAGTTACCGGGGCTGCCCGCGTGGCAAACCATGCTCAAAAGCTTTCCAAATTCTTTGAGTAATC‑AAaaggagaaatcaatatgatcgtttctgacatcgaagctaacgccctcttagagagcgtcactaagttccactgcggggttatctacgac                                                                                                                                                                                                                   <  1:465232‑M1/293‑90 (MQ=255)`  
                                                                                            `gacaggattaaGAAGTTAGGCCAACTGCTGGTTCGTATGTACAACGTGGAAGCCAAGCGACTGAACGATGAGGCTCGTAAAGAGGCCACACAGTCACGCGCTCTGGCGATTCGCTCCAACGAACTGGCTGACAGTGCATCCACTAAAGTTACCGAGGCTGCCCGTGTGGCAAACCAAGCTCAACAGCTTTCCAAATTCTTTGAGTAATCAAAcaggagaaatcaatatgatcgtttctgacatcgaagctaacgccctattagagagcgtcactaagttccactgcggggttatctacgac                                                                                                                                                                                                                   <  1:435196‑M1/296‑90 (MQ=255)`  
                                                                                                 `gATTAAGAAGTTAGGCCAACTGCTGGTTCGTATGTACAACGTGGAAGCCAAGCGACTGAACGATGAGGCTCGTAAAGAGGCCACACAGTCACGCGCTCTGGCGATTCGCTCCAACGAACTGGCTGACAGTGCATCCACTAAAGTTACCGAGGCTGCCCGTGTGGCAAACCAAGCTCAACAGCTTTCCACATTCTTTGAGTAATCAAAcaggagaaatcaatatgatcgtttctgacatcgaagctaacgccctcttagagagcgtcactaagttccactgcgtggttatctacgactactc                                                                                                                                                                                                              >  1:620023‑M1/1‑207 (MQ=255)`  
                                                                                                 `gATTAAGAAGTTAGGCCAACTGCTGGTTCGTATGTACAACGTGGAAGCCAAGCGACTGAACGATGAGGCTCGTAAAGAGGCCACACAGTCACGCGCTCTGGCGATTCGCTCCAACGAACTGGCTGACAGTGCATCCACTAAAGTTACCGAGGCTGCCCGTGTGGCAAACCAAGCTCAACAGCTTTCCAAATTCTTTGAGTAATC‑AAtaggagaaatcaatatgatcgtttctgacctcgaagctaacgccctcttagagagcgtcactaagttccactgcggggttatctccgactactac                                                                                                                                                                                                             >  1:584247‑M1/1‑206 (MQ=255)`  
                                                                                                 `gATTAAGAAGTTAGGCCAACTGCTGGTTCGTATGTACAACGTGGAAGCCAAGCGACTGAACGATGAGGCTCGTAAAGAGGCCACACAGTCACGCGCTCTGGCGATTCGCTCCAACGAACTGGCTGACAGTGCATCCACTAAAGTTACCGAGGCTGCCCGTGTGGCAAACCAAGCTCAACAGCTTTCCAAATTCTTTGAGTAATC‑AAtaggagaaatcaatatgatcgtttctgacatcgaagctaacgccctcttagcgagcgtcactaagttccactgcggggttatctacgacctgtct                                                                                                                                                                                                             >  1:577528‑M1/1‑206 (MQ=255)`  
                                                                                                  `aTTAAGAAGTTAGGCCAACTGCTGGTTCGTATGTACAACGTGGAAGCCAAGCGACTGAACGATGAGGCTCGTAAAGAGGCCACACAGTCACGCGCTCTGGCGATTCGCTCCAACGAACTGGCTGACAGTGCATCCACTAAAGTTACCGAGGCTGCCCGTGTGGCAAACCAAGCTCAACAGCTTTCCAAATTCTTTGAGTAATC‑AAtaggagaaatcaatatgatcgtttctgacatcgaagctaacgccctcttagagagcgtcactaagttccactgcggggttatctacgcctactccc                                                                                                                                                                                                            >  1:333645‑M1/1‑205 (MQ=255)`  
                                                                                                   `ttAAGAAGTTAGGCCAACTGCTGGTTCGTATGTACAACGTGGAAGCCAAGCGACTGAACGATGAGGCTCGTAAAGAGGCCACACAGTCACGCGCTCTGGCGATTCGCTCCAACGAACTGGCTGACAGTGCATCCACTAAAGTTACCGAGGCTGCCCGTGTGGCAAACCAAGCTCAACAGCTTTCCAAATTCTTTGAGTAATC‑AAtaggagaaatcaatatgatcgtttctgacatcgaagctaacgccctcttagagagcgtcactaagttccactgcggggttatctacgactactccac                                                                                                                                                                                                           <  1:231386‑M1/301‑98 (MQ=255)`  
                                                                                                    `tAAGAAGTTAGGCCAACGTCTCGTTCGTATGTACAACGTGGAAGCCAGTCGACTGAACGATGAGGCGCGTAAAGAGGCCACACAGTCACGCGCTCTGGCGATTCGCTCCAACGAACTGGCTGACATTGCATCCACTAAATATACCTAGGCTGCTCGTGTGGCAAACCAAGCTCAACAGCTTTCCAAATTCTTTGAGTAATCAAAcaggagaaatcaatataatcgtttctgacatctaagctatcgccctcttagagagcgtcactaagttccactgcggggttatctacgactactccac                                                                                                                                                                                                           <  1:648549‑M1/301‑98 (MQ=255)`  
                                                                                                       `gaagTTAGGCCAACTGCTGGTTCGTATGTACAACGTGGAAGCCAAGCGACTGAACGATGAGGCTCGTAAAGAGGCCACACAGTCACGCGCTCTGGCGATTCGCTCCAACGAACTGGCTGACAGTGCATCCACTAAAGTTACCGAGGCTGCCCGTGTGGCAAACCAAGCTCAACATCTTTCCAAATTCTTTGAGTAATC‑AAtaggagaaatcaatatgatcgtttctgacatcgaagctaacgccctcttagagagcgtcactaagttccactgcggggttatctacgactactccaccgct                                                                                                                                                                                                       >  1:255024‑M1/1‑200 (MQ=255)`  
                                                                                                             `aGGCCAACGGCTGGTTAGGATGTATGACGTGGAAGCCAAGCGACTGAACGATGAGGCTCGTAAAGAGGCCACACATTCACGCGCTCTGGCGATTCGCTCCAACGAACTGGCTGACAGTGCATCCACTAAATTTACCGAGGCTGCCCGTGTGGCAAACCAAGCTCAACAGCTTTCCAAATGCTTTGAGTAATC‑AAtaggagaaatcaatatgatcgtttctgacatcgaagctaacgccctcttagagagcgtcactaagttccactgcggggttatctacgactactccaccgctgagtac                                                                                                                                                                                                 <  1:141903‑M1/301‑108 (MQ=255)`  
                                                                                                                  `aaCTGCTGGTTCGTATGTACAACGTGGAAGCCAAGCGACTGAACGATGAGGCTCGTAAAGAGGCCACACAGTCACGCGCTCTGGCGATTCGCTCCAACGAACTGGCTGACAGTGCATCCACTAAAGTTACCGAGGCTGCCAGTGTGGCAAACCAAGCTCAACAGCTTTCAAAATTCTTTGAGTAATC‑AAtaggagaaatcaatatgatcgtttctgacatcgaagctaacgccctcttagagagcgtcactaagttccactgcggggttatctacgactactccaccgctgagtacgtaag                                                                                                                                                                                            <  1:59630‑M1/301‑113 (MQ=255)`  
                                                                                                                   `aCTGCTGGTTCGTATGTACAACGTGGAAGCCAAGCGACTGAACGATGAGGCTCGTAAAGAGGCCACACAGTCACGCGCTCTGGCGATTCGCTCCAACGAACTGGCTGACAGTGCATCCACTAAAGTTACCGAGGCTGCCCGTGTGGCAAACCAAGCTCAACAGCTTTCCAAATTCTTTGAGTAATC‑AAtaggagaaatcaatatgatcgtttctgacatcgaagctaacgccctcttagagagcgtcactaagttccactgcggggttatctacgactactccaccgctgagtacgtaagc                                                                                                                                                                                           <  1:494845‑M1/301‑114 (MQ=255)`  
                                                                                                                    `ctgctgGTTCGTATGTACAACGTGGAAGCCAAGCGACTGAACGATGAGGCTCGTAAAGAGGCCACACAGTCACGCGCTCTGGCGATTCGCTCCAACGAACTGGCTGACAGTGCATCCACTAAAGTTACCGAGGCTGCCCGTGTGGCAAACCAAGCTCAACAGCCTTCCAAATTCTTTGAGTAATC‑AAtaggagaaatcaatatgatcgtttctgacatcgaagctaacgccctcttagagagcgtcactaagttccactgcggggttatctacgactactccaccgctgagtacgtaagct                                                                                                                                                                                          <  1:291796‑M1/301‑115 (MQ=255)`  
                                                                                                                      `gctgGTTCGTATGGACAACGTGGAAGCCAAGCTACTGAACGATGAGGCTCGTAAAGAGGCCACACAGTCACGCGCTCTGGCGATTCGCTCCAACGAACTGGCTGACAGTGCATCCACTAAAGTTACCGAGGCTGCCCGTGTGGCAAACCAAGCTCAACAGCTTTCCAAATTCTTTGAGTAATC‑AAtaggagaaatcaatatgatcgtttctgacatcgaagctaacgccctcttagagagcgtcactaagttccactgcggggttatctacgactactccaccgctgagtacgtaagctac                                                                                                                                                                                        <  1:551584‑M1/301‑117 (MQ=255)`  
                                                                                                                             `cGTATGTACAACGTGGAAGCCAAGCGACTGAACGATGAGGCTCGTAAAGAGGCCACACAGTCACGCGCTCTGGCGATTCGCTCCAACGAACTGGCTGACAGTGCATCCACTAAAGTTACCGAGGCTGCCCGTGTGGCAAACCAAGCTCAACAGCTTTCCAAATTCTTTGAGTAATT‑AAtaggagaaatcaatatgatcgtttctgacatcgaagctaacgccctcttagagagtgtcactaagttccactgcggggttatctacgactactccatcgctgagtacgtaagctaccgtccga                                                                                                                                                                                 >  1:52047‑M1/1‑178 (MQ=255)`  
                                                                                                                             `cGTATGTACAACGTGGAAGCCAAGCGACTGAACGATGAGGCTCGTAAAGAGGCCACACAGTCACGCGCTCTGGCGATTCGCTCCAACGAACTGGCTGACAGTGCATCCACTAAAGTTACCGAGGCTGCCCGTGTGGCAAACCAAGCTCAACAGCTTTCCAAATTCTTTGAGTAATC‑AAtaggagaaatcaatatgatcgtttctgacatcgaagctaacgccctcttagagagcgtcactaagttccactgcggggttatctacgactactccaccgctgagtacgtaagctaccgtccgg                                                                                                                                                                                 >  1:236498‑M1/1‑178 (MQ=255)`  
                                                                                                                             `cGTATGTACAACGTGGAAGCCAAGCGACTGAACGATGAGGCTCGTAAAGAGGCCACACAGTCACGCGCTCTGACGATTCGCTCCAACGAACTGGCTGACAGTGCATCCACTAAAGTTACCGAGGCTGCCCGTGTGGCAAACCAAGCTCAACAGCTTTCCAAATTCTTTGAGTAATC‑AAtaggagaaatcaatatgatcgtttctgacatcgaagctaacgccctcttagagagcgtcactaagttccactgcggggttatctacgactactccaccgctgagtacgtaagctaccgtccga                                                                                                                                                                                 >  1:543152‑M1/1‑178 (MQ=255)`  
                                                                                                                               `tATGTACAACGTGGAAGCCAAGCGACTGAACGATGAGGCTCGTAAAGAGGCCACACAGTCACGCGCTCTGGCGATTCGCTCCAACGAACTGGCTGACAGTGCATCCACTAAAGTTACCGAGGCTGCCCGTGTGGCAAACCAAGCTCAACAGCTTTCCAAATTCTTTGAGTAATCAAAcaggagaaaccattatgatcgtttctgacatcgaagctaacgcactcttagagagcgtcactaagttccactccggggttatctacgactactccaccgctgagtacgtaagctaccgtccgag                                                                                                                                                                                >  1:67545‑M1/1‑177 (MQ=255)`  
                                                                                                                                  `gTAAAACGTGGAAACCAAGCGACTGAACTATGAGGCTCGTAAAGAGGCCACACAGTCACGCGCTCTGGCGATTCGCTCCAACGAACTGGCTGACAGTGCATCCAATAAAGTTACCGAGGCTGCCCGTGGGGCAAACCAAGCTCAACAGCTTTCCAAATTCTTTGAGTAATC‑AAtaggagaaatcaatatgatcgtttctgacatcgaagctaacgccctcttagagagcgtcactaagttccactgcggggttatctacgactactccaccgctgagtacgtaagctaccgtccgagtgac                                                                                                                                                                            <  1:243804‑M1/301‑129 (MQ=255)`  
                                                                                                                                      `aaCGTGGAAGCCAAGCGACTGAACGATGAGGCTCGTAAAGAGGCCACACAGTCACGCACTCTGGCGATTCGCTCCAACGAACTGGCTGACAGTGCATCCACTAAAGTTACCGAGGCTGCCCGTGTGGCAAACCAAGCTCAACAGCTTTCCAAATTCTTTGAGTAATCAAAcaggagaaatcaatatgatcgtttctgacatcgaagctaacgccctcttagagagcgtcactaagttccactgcggggttatctacgactactccaccgctgagtacgtaagctaccgtccgagtgacttc                                                                                                                                                                         <  1:388614‑M1/301‑132 (MQ=255)`  
                                                                                                                                          `tGGAAGCCAAGCGACTGAACGATGAGGCTCTTAAAGAGGCCACACAGTCACGCGCTCTGGCGATTCGCTCAAACGAACTGGCTGACAGTGCATCCACTAAAGTTACCGAGGCTGCCCGTGTGGCAAACCAAGCTCAACAGCTTTCCAAATTCTTTGAGTAATCAAAcaggagaaatcaatatgatcgtttctgacatcgaagctaacgccctcttagagagcgtcactaagttccgctgcggggttatctacgactactccaccgctgagtacgtaagctaccgtccgagtgacttcggtg                                                                                                                                                                     <  1:321616‑M1/301‑136 (MQ=255)`  
                                                                                                                                               `gCCAAGCGACTGAACGATGAGGCTCGTAAAGAGGCCACACAGTCACGCGCTCTGGCGATTCGCTCCAACGAACTGGCTGACAGTGCATCCACTAAAGTTACCGAGGCTGCCCGTGTGGCAAACCAAGCTCAACAGCTTTCCAAATTCTTTGAGTAATC‑AAtaggagaaatcaatatgatcgtttctgacatcgaagctaacgccctcttagagagcgtcactaagttccactgcggggttatctacgactactccaccactgagtacgtaagctaccgtccgagtgacttcggtgcgtatc                                                                                                                                                               <  1:171901‑M1/301‑142 (MQ=255)`  
                                                                                                                                                  `aaGCGACTGAACGATGAGGCTCGTAAAGAGGCCACACAGTCACGCGCTCTGGCGATTCGCTCCAACGAACTGGCTGACAGTGCATCCACTAAAGTTACCGAGGCTGCCCGTGTGGCAAACCAAGCTCAACAGCTTTCCAAATTCTTTGAGTAATCAAAcaggagaaatcaatatgatcgtttctgacatcgaagctaacgccctcttagagagcgtcactaagttccactgcggggttatctacgactactccaccgctgagtacgtaagctaccgtccgagtgacttcggtgcgtagctg                                                                                                                                                             <  1:346767‑M1/301‑144 (MQ=255)`  
                                                                                                                                                    `gCGACTGAACGATGAGGCTCGTAAAGAGGCAACCCAGTCACGCGCTCTGGCGATTCGCTCCAACGAACTGGCTGACAGTGCATCCACTAAAGTTACCGAGGCTGCCCGTGTGGCAAACCAAGCTCAACAGCTTTCCAAATTCTTTGAGTAATCAAAcaggagaaatcaatatgatcgtttctgacatcgaagctaacgccctcttagagagcgtcactaagttccactgcggggttatctacgactactccaccgctgagtacgtaagctaccgtccgagtgacttcggtgcgtatctgga                                                                                                                                                           <  1:154371‑M1/301‑146 (MQ=255)`  
                                                                                                                                                          `gAACGATGAGGCTCGTAAAGAGGCCACACAGTCACGCGCTCTGGCGATTCGCTCCAACGAACTGGCTGACAGTGCATCCACTAAAGTTACCGAGGCTGCCCGTGTGGCAAACCAAGCTCAACAGCTTTCCAAATTCTTTGAGTAATC‑AAtaggagaaatcaatatgatcgtttctgacatcgaagctaacgccctcttggagagcgtcactaagttccactgcggggttatctacgactactccaccgctgagtacgtaagctaccgtccgcgtgacttcggtgcgtatctggatgcgctg                                                                                                                                                    >  1:16485‑M1/1‑149 (MQ=255)`  
                                                                                                                                                               `aTGAGGCTCGTAAAGAGGCCACACAGTCACGCGCTCTGGCGATTCGCTCCAACGAACTGGCTGACAGTGCATCCACTAAAGTTACCGAGGCTGCCCGTGTGGCAAACCAAGCTAAACAGCTTTCCAAATTCTTTGAGTAATC‑AAtaggagaaatcaatatgatcgtttctgacatcgaagctaacgccctcttagagagcgtcactaagttccactgcggggttatctacgactactccaccgctgagtacgtaagctaccgtccgagtgacttcggtgcgtatctggatgcgctggaagc                                                                                                                                               >  1:590681‑M1/1‑144 (MQ=255)`  
                                                                                                                                                                               `ggCCACACAGTCACGCGCTCTGGCGATTCGCTCCAACGAACTGGCTGACAGTGCATCCACTAAAGTTACCGAGGCTGCCCGTGTGGCAAACCAAGCTCAACAGCTTTCCAAATTCTTTGAGTAATCAAAcaggagaaatcaatatgatcgtttctgacatcgaagctaacgccctcttagagagcgtcactaagttccactgcggggttatctacgactactccaccgctgagtccgtaagctaccgtccgagtgacttcggtgcgtatctggatgcactggaagccgaggttgcacgagg                                                                                                                                >  1:134212‑M1/1‑129 (MQ=255)`  
                                                                                                                                                                                `gCCACACAGTCACGCGCTCTGGCGATTCGCTCCAACGAACTGGCTGACCGTGCATCCACGAAAGTTACCGAGGCTGCCCGTGTGGCAAACCAAGCTCAACAGCTTTCCAAATTCTTTGAGTAATCAAAcaggagaaatcaatatgatcgtttctgacatcgaagctaacgccctcttagagagcgtcactaagttccactgcggggttatctacgactactccaccgctgagtacgtaagctaccgtccgagtgacttcggtgcgtatctggatgcgctggaagccgaggttgcacgaggc                                                                                                                               <  1:214761‑M1/301‑174 (MQ=255)`  
                                                                                                                                                                                `gCCACACAGTCACGCGCTCTGGCGATTCGCTCCAACGAACTGGCTGACAGTGCATCCACTAAAGTTACCGAGGCTGCCCGTGTGGCAAACCAAGCTCAACAGCTTTCCAAATTCTTTGAGTAATCAAAcaggagaaatcaatatgatcgtttctgacatcgaagctaacgccctcttagagagcgtcactaagttccactgcggggttatctacgactactccaccgctgagtacgtaagctaccgtccgagtgacttcggtgcgtatctggatgcgctggaagccgaggttgcacgaggc                                                                                                                               <  1:122282‑M1/301‑174 (MQ=255)`  
                                                                                                                                                                                   `acacaGTCACGCGCTCTGGCGATTCGCTCCAACGAACTGGCTGACAGTGCATCCACTAAAGTTACCGAGGCTGCCCGTGTGGCAAACCAAGCTCAACAGCTTTCCAAATTCTTTGAGTAATC‑AAtaggagaaatcaatatgatcgtttctgacatcgaagctaacgccctcttagagagcgtcactaagttccactgcggggttatctacgactactccaccgctgagtacgtaagctaccgtccgagtgacttcggtgcgtatctggatgcgctggaagccgaggttgcccgaggcggtc                                                                                                                           >  1:363556‑M1/1‑124 (MQ=255)`  
                                                                                                                                                                                    `aacagTCACGCGCACTGGCGATTCGCTCCAACGAACTGGCTGACAGTGCATCCACTAAAGTTACCGAGGCTGCCCGTGTGGCAAACCAAGCTCAACAGCTTTCCAAATTCTTTGAGTAATC‑AAtaggagaaatcaatatgatcgtttctgacatcgaagctaacgccctcttagagagcgtcactaagttccactgcggggttatctacgactactccaccgctgagtacgtaagctaccgtccgagtgacttcggtgcgtatctggatgcgctggaagccgaggttgcacgaggcggtct                                                                                                                          <  1:641646‑M1/300‑179 (MQ=255)`  
                                                                                                                                                                                        `gTCACGCGCTCTGGCGATTCGCTCCAACGAACTGGCTGACAGTGCATCCACTAAAGTTACCGAGGCTGCCCGTGTGGCAAACCAAGCTCAACAGCTTTCCAAATTCTTTGAGTAATCAAAcaggagaaatcaatatgatcgtttctgacatcgaagctaacgccctcttagagagcgtcactaagttccactgcggggttatctacgactactccaccgctgagtacgtaagctaccgtccgagtgacttcggtgcgtatctggatgcgctggaagccgaggttgcacgaggcggtcttat                                                                                                                       <  1:36900‑M1/301‑182 (MQ=255)`  
                                                                                                                                                                                         `tCACGCGCTCTGGCGATTCGCTCCAACGAACTGGCTGACAGTGCATCCACTAAAGTTACCGAGGCTGCCCGTGTGGCAAACCAAGCTCAACAGCTTTCCAAATTCTTTGAGTAATC‑AAtaggagaaatcaatatgatcgtttctgacatcgaagctaacgccctcttagagagcgtcactaagttccactgcggggttatctacgactactccaccgctgagtacgtaagctaccgtccgagtgacttcggtgcgtatctggatgcgctggaagccgaggttgcacgaggcggtcttattg                                                                                                                     <  1:557304‑M1/301‑184 (MQ=255)`  
                                                                                                                                                                                         `gcaCGCGCTCTGGCGATTCGCTCCAACGAACTGGCTGACAGTGCATCAACTAAAGTTACCGAGGCTGACCGTGTGGAAAACCAAGCTCAACAGCTTTCCAAATTCTTTGAGTAATC‑AAtaggagaaagcaatatgatcgtttctgacatcgaagctaacgccctcttagagagcgtcactaagttccactgcggggttatctacgactactccaccgctgagtacgtaagctaccgtccgagtgacttcggtgcgtatctggatgcgctggaagccgaggttgcacgaggcggtcttattg                                                                                                                     <  1:184516‑M1/300‑184 (MQ=255)`  
                                                                                                                                                                                                  `ctGGTGATTCGCTCCAACGAACTGGCTGACAGTGCATCCACTAAAGTTACCGAGGCTGCCCGTGTGGCAAACCAAGCTCAACAGCTTTCCAAATTCTTTGAGTAATC‑AAtaggagaaatcaatatgatcgtttctgacatcgaagctaacgccctcttagagagcgtcactaagttccactgcggggttatctacgactactccaccgctgagtacgtaagctaccgtccgagtgacttcggtgcgtatctggatgcgctggaagccgaggttgcacgaggcggtcttattgtgttccaca                                                                                                            >  1:148601‑M1/1‑109 (MQ=255)`  
                                                                                                                                                                                                  `ctGGCGATTCGCTCCAACGAACTGGCTGACAGTGCATCCACTAAAGTTACCGAGGCTGCCCGTGTGGCAAACCAAGCTCAACAGCTTTCCAAATTCTTTGAGTAATCAAAcaggagaaatcaatatgatcgtttctgacatcgaagctaacgccctcttatagagcgtcactaagttccactgcggggttatctacgactactccaccgctgagtacgtaagctaccgtccgagtgacttcggtgcgtatctggatgcgctggaagccgaggttgcacgaggcggtcttattgtgttccac                                                                                                             <  1:12946‑M1/301‑192 (MQ=255)`  
                                                                                                                                                                                                    `ggCGATTCGCTCCAACGAACTAGCTGACAGTGCATCCACTAAAGTTACCGAGGCTGCCCGTGTGGCAAACCAAGCTCAACAGCTTTCCAAATTCTTTGAGTAATCAAAcaggagaaatcaatatgatcgtttctgacatcgaagctaacgccctcttagagagcgtcactaagttccactgcggggttatctacgactactccaccgctgagtacgtaagctaccgtacgagtgacttcggtgcgtatctggatgcgctggaagccgaggttgcacgaggcggtcttattgtgttccacaa                                                                                                           <  1:45332‑M1/301‑194 (MQ=255)`  
                                                                                                                                                                                                                    `aaacTGGCTGACAGTGCATCCACTAAAGTTACCGAGGCTGCCCGTGTGGCAAACCAAGCTCAACAGCTTTCCAAATTCTTTGAGTAATC‑AAtagtagaaatcaatatgatcgtttctgacatcgaagctaacgccctcttagagagcgtcactaagttccactgcggggttatctacgactactccaccgctgagtacgtaagctaccgtccgagtgacttcggtgcgtatctggatgcgctggaagccgaggttgcacgaggcggtcttattgtgttccacaacggtcacaagtatgacg                                                                                          <  1:457944‑M1/300‑211 (MQ=255)`  
                                                                                                                                                                                                                     `aaCTGGCTGACAGTGCATCCACCAAAGTTACCGAGGCTGCCCGTGTGGCAAACCAAGCTCAACAGCTTTCCAAATTCTTTGAGTAATCAAAgaggagaaatcaatatgttcgtttctgacatcgaagctaacgccctcttagagagcgtcactaagttccactgcggggttatctacgactactccaccgctgagtacgtaagctaccgtccgagtgacttcggtgcgtatctggatgcgctggaagccgaggttgcacgaggcggtcttattgtgttccacaacggtcacaagtatgacg                                                                                          <  1:190481‑M1/301‑211 (MQ=255)`  
                                                                                                                                                                                                                       `cTGGCTGACAGTGCATCCACTAAAGTTACCGAGGCTGCCCGTGTGGCAAACCAAGCTCAACAGCTTTCCAAATTCTTTGAGTAATCAAAcaggagaaatcaatatgatcgtttctgacatcgaagctaacgccctcttagagagcgtcactaagttccactgcggggttatctacgactactccaccgctgagtacgtaagctaccgtccgagtgacttcggtgcgtatctggatgcgctggaagccgaggttgcacgaggcggtcttattgtgttccacaacggtcacaagtatgactgt                                                                                        >  1:92985‑M1/1‑89 (MQ=255)`  
                                                                                                                                                                                                                         `ggCTTACAGTGCATCCACTAAAGTTACCGAGGCTGCCCGTGTGGCAAACCAAGCTCAACAGCTTTCCAAATTCTTTGAGTAATC‑AAtaggagaaatcaatatgatcgtttctgacatcgcagctaacgccctcttagagagcgtcactaagttccactgcggggttctctacgactactccaccgctgagtacgtaagctaccgtccgagtgacttcggtgcgtatctggatgcgctggaagccgaggttgcacgaggcggtcttattgtgttccacaccgttcccaagtatgacgttcct                                                                                     >  1:525022‑M1/1‑86 (MQ=255)`  
                                                                                                                                                                                                                         `ggCTGACAGTGCATCCACTAAAGTTACCGAGGCTGCCCGTGTGGCAAACCAAGCTCAACAGCTTTCCAAATTCTTTGAGTAATCAAAcaggagaaatcaatatgatcgtttctgacatcgaagctaacgccctcttagagagcgtcactaagttccactgcggggttatctacgactactccaccgctgagtacgtaagctaccgtccgagtgacttcggtgcgtatctggatgcgctggaagccgaggttgcacgaggcggtcttattgtgttccacaacggtcacaagtatgacgttcc                                                                                      >  1:411290‑M1/1‑87 (MQ=255)`  
                                                                                                                                                                                                                         `ggCTGACAGTGCATCCACTAAAGTTACCGAGGCTGCCCGTGTGGCAAACCAAGCTAAACAGCTTTCCAAATTCTTTGAGTAATCAAAcaggagaaatcaatatgatcgtttctgacatcgaagctaacgccctcttagagagcgtcactaagttccactgcggggttatctccgactactccaccgctgagtacgtaagctaccgtccgagtgacttcggtgcgtatctggatgcgctggaagccgaggttgcacgaggcggtcttattgtgttccacaacggtcacaagtatgacgttcc                                                                                      >  1:659510‑M1/1‑87 (MQ=255)`  
                                                                                                                                                                                                                             `gACAGTGCATCCACTAAAGTTACCGAGGCTGCCCGTGTGGCAAACCAAGCTCAACAGCTTTCCAAATTCTTTGAGTAATC‑AAtaggagaaatcaatatgatcgtttctgacatcgaagctaacgccctcttagagagcgtcactaagttccactgcggggttatctacgactactccaccgctgagtacgtaagctaccgtccgagtgacttcggtgcgtatctggatgcgctggaagccgaggttgcacgaggcggtcttattgtgttccacaacggtcacaagtatgacgttcctgcat                                                                                 <  1:304367‑M1/301‑220 (MQ=255)`  
                                                                                                                                                                                                                             `aaagagacagccactaaagttaCCGAGGCTGCCCGTGTGGCAAACCAAGCTCAACAGCTTTACAAATTCGTTGAGTAATC‑AAtaggagaaatcaatatgatcgtttctgacatcgaagctaacgccctcttagagagcgtcactaagttccactgcggggttatctacgactactccaccgctgagtacgtaagctaccgtccgagtgacttcggtgcgtatctggatgcgctggaagccgaggttgcacgaggcggtcttattgtgttccacaacggtcacaagtatgacgttcctgcat                                                                                 <  1:490439‑M1/291‑220 (MQ=255)`  
                                                                                                                                                                                                                                 `gTGCATCCACTAAAGTTACCGAGGTTGCCCGTGTGGCAAACCAAGCTCAACAGCTTTCCAAATTCTTTGAGTAATCAAAcaggagaaatcaatatgatcgtttctgacatcgaagctaacgccctcttagagagcgtcactaagttccactgcggggttatctacgactactccaccgctgagtacgtaagctaccgtccgagtgacttcggtgcgtatctggatgcgctggaagccgaggttgcacgaggcggtcttattgtgttccacaacggtcacaagtatgacgttcctgcattga                                                                              >  1:601680‑M1/1‑79 (MQ=255)`  
                                                                                                                                                                                                                                                                 `gtgGCAAACCAAGCTCAACAGCTTTCCAAATTCTTTGAGTAATCAAAcaggagaaatcaatatgatcgtttctgacatcgaagctaacgccctcttagagagcgtcactaagttccactgcggggttatctacgactactccaccgctgagtacgtcagctaccgtccgagtgacttcggtgcgtatctggatgcgctggaagccgaggttgcacgaggcggtcttattgtgttccacaacggtcacaagtatgacgttcctgcattgacaaaactggcaaagttgcaatttacccgcgag                                              >  1:653460‑M1/1‑47 (MQ=255)`  
                                                                                                                                                                                                                                                                       `aaCCAAGCTCAACAGCTTTCCAAATTCTTTGAGTAATC‑AAtaggagaaatcaatatgatcgtttctgacatcgaagctaacgccctcttagagagcgtcactaagttccactgcggggttatctacgactactccaccgctgagtacgtaagctaccgtccgagtgacttcagtgcgtatctggatgcgctggaagccgaggttgcacgaggcggtcttattgtgttccacaacggccacaagtatgacgttcctgcattgaccaaactggcaaagttgcacttgaaccgagagttccacc                                       >  1:399889‑M1/1‑40 (MQ=255)`  
                                                                                                                                                                                                                                                                                 `tgtataagagacagttctttgagtaatcaaAcaggagaaatcaatatgatcgtttctgacatcgaagctaacgccctcttagagagcgtcactaagttccactgcggggttatctacgactactccaccgctgagtacgtaagctaccgtccgagtgacttcggtgcgtatctggatgcgctggaagccgaggttgcacgaggcggtcttattgtgttccacaacggtcacaagtatgacgttcctgcattgaccaaactggcaaagttgcaattgaaccgagagttccaccttcctcgtg                              <  1:24844‑M1/287‑271 (MQ=255)`  
                                                                                                                                                                                                                                                                                          `ccAAATTCTTTGAGTAATC‑AAtaggagaaatcaatatgatcgtttctgacatcgaagctaacgccctcttagagagcgtcactaagttccactgcggggttatctacgactactccaccgctgagtacgtaagctaccgtccgagtgacttcggtgcgtatctggatgcgctggaagccgaggttgcacgaggcggtcttattgtgttccacaacggtcacaagtatgacgttcctgcattgaccaaactggcaaagttgcaattgaaccgagagttccaccttcctcgtgagaactgtat                    <  1:477035‑M1/301‑281 (MQ=255)`  
                                                                                                                                                                                                                                                                                               `ttCTTTGAGTAATCAAAcaggagaaatcaatatgatcgtttctgtcatcgaagctaacgccctcttagagagcgtgactaagttccactgcggggttatctacgactactccaccgctgagtacgtaagctaccgtccgagtgacttctgtgcgtatctggatgcgctggaagccgaggttgcacgaggcggtcttattgtgttccacaacggtcacaagtatgacgttcctgcattgaccaaactggcaaagttgcaattgaaccgagagttccaccttcctcgtgagaactgtattgac                <  1:508082‑M1/301‑285 (MQ=255)`  
                                                                                                                                                                                                                                                                                               `ttCTTTGAGTAATC‑AAtaggagaaatcaatatgatcgtttctgacatcgaagctaacgccctcttagagatcgtcactaagttccactgcggggttatctacgactactccaccgctgagtacgtaagctaccgtccgagtgacttcggtgcgtatctggatgcgctggaagccgaggttgcacgaggcggtcttattgtgttccacaacggtcacaagtatgacgttcctgcattgaccaaactggcaaagttgcaattgaaccgagagttccaccttcctcgtgctgtctcttatacac               >  1:562226‑M1/1‑16 (MQ=255)`  
                                                                                                                                                                                                                                                                                                  `tttGAGTAATCAAAcaggagaaaccattatgatcgtttctgacatcgaagctaacgccctcttagagagcgtcactaagttccacggcggggttatctacgactactccaccgctgagtacgtaagctaccgtccgagtgacttcggtgcgtatctggatgcgctggaagccgaggttgcacgaggcggtcttattgtgttccacaacggtcacaagtatgacgttcctgcattgaccaaactggcaaagttgcaattgaacctagagttccaccttcctcgtgagaactgtattgacacc             <  1:105401‑M1/301‑288 (MQ=255)`  
                                                                                                                                                                                                                                                                                                     `gAGTAATC‑AAtaggagaaatcaatatgatcgtttctgacatcgaagctaacgccctcttagagagcgtcactaagttccactgcggggttatctacgactactccaccgctgagtacgtaagctaccgtccgagtgacttcggtgcgtatctggatgcgctggaagccgaggttgcacgaggcggtcttattgtgttccacaacggtcacaagtatgacgttcctgcattgaccaaactggcaaagttgcaattgaaccgagagttccaccttcctcgtgagaactgtatttacactgtcc         >  1:359440‑M1/1‑10 (MQ=255)`  
                                                                                                                                                                                                                                                                                                        `tAATC‑AAtaggataaatcaatatgatcgtttctgacttcgaagctaacgccctcttagagagcgtcactacgttccactgcggtgttatctacgactactccaccgctgagtacgtaagctaccgtccgagtgacttcggtgcgtatctggatgcgctggaagtcgaggttgcacgaggcggtcttattgtgttccacaacggtcacaagtatgacgttcctgcattgaccaaactggcaaagttgcaattgaaccgagagttccaccttcctcgtgagaactgtattgacacccttgtgt      <  1:297764‑M1/301‑295 (MQ=255)`  
                                                                                                                                                                                                                                                                                                        `tAATC‑AAtaggagaaatcaatatgatcgtttctgacatcgaagctaacgccctcttagagagcgtcactaagttccactgcggggttatctacgactactccaccgctgagtacgtaagctaccgtccgagtgacttcggtgcgtatctggatgcgctggaagccgaggttgcacgaggcggtcttattgtgttccacaacggtcacaagtatgacgttcctgcattgaccaaactggcaaagttgcaattgaaccgagagttccaccttcctcgtgagaactgtattgacaccattgtgt      <  1:310166‑M1/301‑295 (MQ=255)`  
                                                                                                                                                                                                                                                                                                          `aTC‑AAtaggaggaatcaatatgatcgtttctgacatcgaagctaacgccctcttagagagcgtcactaagttccactgcggggttatctacgactactccaccgctgagtacgtaagctaccgtccgagtgacttcggtgcgtatctggatgcgctggaagccgaggttgcacgaggcggtcttattgtgttccacaacggtcacaagtatgacgttcctgcattgaccaaactggcaaagttgcaattgaaccgagagttccaccttcctcgtgagaactgtattgacactgtctcttat    >  1:515177‑M1/1‑5 (MQ=255)`  
                                                                                                                                                                                                                                                                                                          `aTC‑AAtaggagaaatcaatatgatcgtttctgacatcgaagctaacgccctcttagagagcgtcactaagttccactgcggggttatctacgactactccaccgctgagtacgtaagctaccgtccgagtgacttcgatgcgtatctggatgcgctggaagccgaggttgcacgaggcggtcttattgtgttccacaacggtcacaagtatgacgttcctgcattgaccaaactggcaaagttgcaattgaaccgagagttccaccttcctcgtgagaactgtattgacacccttgtgctg    >  1:285901‑M1/1‑5 (MQ=255)`  
                                                                                                                                                                                                                                                                                                           `tC‑AAtaggagaaatcaatatgatcgtttctgacatcgaagctaacgccctcttagagagcgtcactaagttccactgcggggttatctacgactactccaccgctgagtacgtaagctaccgtccgagtgacttcggtgcgtatctggatgcgctggaagccgaggttgcacgaggcggtcttattgtgttccacaacggtcacaagtatgacgttcctgcattgaccaaactggcaaagttgcaattgaaccgagagttccaccttcctcgtgagaacctgtctcttatacacatctccc   >  1:624720‑M1/1‑4 (MQ=255)`  
                                                                                                                                                                                                                                                                                                            `c‑AAtaggagaaatcaatatgatcgtttctgacatcgaagctaacgccctcttagagagcgtcactaagttccactgcggggttatctacgactactccaccgctgagtacgtaagctaccgtccgagtgacttcggtgcgtatctggatgcgctggaagccgaggttgcacgaggcggtcttattgtgttccacaacggtcacaagtatgacgttcctgcattgaccaaactggcaaagttgcaattgaaccgagagttccaccttcctcgtgagaactgtattgacaccctgtctcttat  >  1:339875‑M1/1‑3 (MQ=255)`  
                                                                                                                                                                                                                                                                                                            `c‑AAtaggagaaatcaatatgatcgtttctgacatcgaagctaacgccctcttagagagcgtcactaagttccactgcggggttatctacgactactccaccgctgagtacgtaagctaccgtccgagtgacttcggtgcgtatctggatgcgctggaagccgaggttgcacgaggcggtcttattgtgttccacaacggtaacaagtatgacgttcctgcattgaccaaactggcaaagttgcaattgaaccgagagttccaccttcctcgtgagaactgtattgacacccttgtgttgtt  >  1:530437‑M1/1‑3 (MQ=255)`  
                                                                                                                                                                                                                                                                                                            `c‑AAtaggagaaatcaatatgatcgtttctgacatcgaagctaacgccctcttagagagcgtcactaagttccactgcggggttatctacgactactccaccgctgagtacgcaagctaccgtccgagtgacttcggtgcgtatctggatgcgctggaagccgaggttgcacgaggcggtcttattgtgttccacaacggtcacaagtatgacgttcctgcattgaccaaactggcaaagttgcaattgaaccgagagttccaccttcctcgtgagaactgtattgacacccttgtgtctgt  >  1:260709‑M1/1‑3 (MQ=255)`  

                                                                                                                                                                                                                                                                                                             `|`                                                                                                                                                                                                                                                                                                              
`CAGGATTCTTGATGACTTTCCAGACGACTACGAGAAGTTTCGCTGGAGAGTCCCATTCTAATACGACTCACTAAAGGAGACACACCATGTTCAAACTGATTAAGAAGTTAGGCCAACTGCTGGTTCGTATGTACAACGTGGAAGCCAAGCGACTGAACGATGAGGCTCGTAAAGAGGCCACACAGTCACGCGCTCTGGCGATTCGCTCCAACGAACTGGCTGACAGTGCATCCACTAAAGTTACCGAGGCTGCCCGTGTGGCAAACCAAGCTCAACAGCTTTCCAAATTCTTTGAGTAATC‑AAACAGGAGAAACCATTATGTCTAACGTAGCTGAAACTATCCGTCTATCCGATACAGCTGACCAGTGGAACCGTCGAGTCCACATCAACGTTCGCAACGGTAAGGCGACTATGGTTTACCGCTGGAAGGACTCTAAGTCCTCTAAGAATCACACTCAGCGTATGACGTTGACAGATGAGCAAGCACTGCGTCTGGTCAATGCGCTTACCAAAGCTGCCGTGACAGCAATTCATGAAGCTGGTCGCGTCAATGAAGCTATGGCTATCCTCGACAAGATTGATAACTAAGAGTGGTATCCTC  >  NC_001604.1/13266‑13866`

  

Alignment Legend

Aligned base mismatch/match (shaded by quality score): `ATCG/ATCG` `< 3 ≤` `ATCG/ATCG` `< 10 ≤` `ATCG/ATCG` `< 22 ≤` `ATCG/ATCG` `< 34 ≤` `ATCG/ATCG` `< 38 ≤` `ATCG/ATCG`

Unaligned base: `atcg`    Masked matching base: `atcg`    Alignment gap: `‑`    Deleted base: `‑`
